# The Effect of Ca^2+^, Lobe-Specificity, and CaMKII on CaM Binding to Na_V_1.1

**DOI:** 10.3390/ijms19092495

**Published:** 2018-08-23

**Authors:** Jianing Li, Zhiyi Yu, Jianjun Xu, Rui Feng, Qinghua Gao, Tomasz Boczek, Junyan Liu, Zhi Li, Qianhui Wang, Ming Lei, Jian Gong, Huiyuan Hu, Etsuko Minobe, Hong-Long Ji, Masaki Kameyama, Feng Guo

**Affiliations:** 1 Department of Pharmaceutical Toxicology, School of Pharmacy, China Medical University, Shenyang 110122, China; Ariene77687@outlook.com (Ji.L.); fengrui527@163.com (R.F.); rwygao@m.kufm.kagoshima-u.ac.jp (Q.G.); m18602444860_1@163.com (Ju.L.); lizhi0824@live.com (Z.L.); WQH91225@163.com (Q.W.); leiming@swmu.edu.cn (M.L.); hyhu@cmu.edu.cn (H.H.); 2 Department of Neurobiology, Harvard Medical School, Boston, MA 02115, USA; zhiyi7566@live.cn; 3 Department of Physiology, Graduate School of Medical and Dental Sciences, Kagoshima University, Kagoshima 890-8544, Japan; jjxuk@m3.kufm.kagoshima-u.ac.jp (J.X.); mimiben@m3.kufm.kagoshima-u.ac.jp (E.M.); 4 Department of Ophthalmology, Stanford University School of Medicine, Palo Alto, CA 94305, USA; tomasz.boczek@umed.lodz.pl; 5 Department of Clinical Pharmacy, School of Life Science and Biopharmaceutics, Shenyang Pharmaceutical University, Shenyang 110016, China; fanxing1230@163.com; 6 Department of Cellular and Molecular Biology, University of Texas Health Science Center at Tyler, Tyler, TX 75708, USA; james.ji@uthct.edu

**Keywords:** Ca^2+^, CaM, CaMKII, Na_V_1.1, IQ domain

## Abstract

Calmodulin (CaM) is well known as an activator of calcium/calmodulin-dependent protein kinase II (CaMKII). Voltage-gated sodium channels (VGSCs) are basic signaling molecules in excitable cells and are crucial molecular targets for nervous system agents. However, the way in which Ca^2+^/CaM/CaMKII cascade modulates Na_V_1.1 IQ (isoleucine and glutamine) domain of VGSCs remains obscure. In this study, the binding of CaM, its mutants at calcium binding sites (CaM_12_, CaM_34_, and CaM_1234_), and truncated proteins (N-lobe and C-lobe) to Na_V_1.1 IQ domain were detected by pull-down assay. Our data showed that the binding of Ca^2+^/CaM to the Na_V_1.1 IQ was concentration-dependent. ApoCaM (Ca^2+^-free form of calmodulin) bound to Na_V_1.1 IQ domain preferentially more than Ca^2+^/CaM. Additionally, the C-lobe of CaM was the predominant domain involved in apoCaM binding to Na_V_1.1 IQ domain. By contrast, the N-lobe of CaM was predominant in the binding of Ca^2+^/CaM to Na_V_1.1 IQ domain. Moreover, CaMKII-mediated phosphorylation increased the binding of Ca^2+^/CaM to Na_V_1.1 IQ domain due to one or several phosphorylation sites in T1909, S1918, and T1934 of Na_V_1.1 IQ domain. This study provides novel mechanisms for the modulation of Na_V_1.1 by the Ca^2+^/CaM/CaMKII axis. For the first time, we uncover the effect of Ca^2+^, lobe-specificity and CaMKII on CaM binding to Na_V_1.1.

## 1. Introduction

Voltage-gated sodium channels (VGSCs) are basic signaling molecules in excitable cells and are molecular targets for local anesthetic agents and antiepileptic agents [1,2]. So far, ten isoform members have been identified—Na_V_1.1-Na_V_1.9 and NaX—forming the VGSCs superfamily [3,4] in which Na_V_1.1 is widely expressed in cell bodies and axon initial segments of neurons in the brains [5,6,7,8,9,10,11,12,13]. A sequence within C-terminal of Na_V_1.1 contains a classical calmodulin (CaM)-binding IQ (isoleucine and glutamine) domain [14,15,16,17], which is involved in Ca^2+^ signal transduction and alters the activity in response to changes in free Ca^2+^ concentration ([Ca^2+^]). All ten isoforms of VGSCs contain a unique IQ domain [14,15].

It has previously been demonstrated that Ca^2+^ plays a crucial role in the physiology of mammalians and that it is involved in the regulation of many intracellular processes ranging from gene transcription to neurotransmitter release [18,19,20]. The intracellular free [Ca^2+^] increases from ~10^−7^ M during resting state and up to ~10^−3^ M during active state [21,22]. It has been found that Ca^2+^/CaM modulates VGSCs activity [12,23,24], but the molecular mechanism of how Ca^2+^/CaM binds to Na_V_1.1 is still unclear.

The CaM molecule is composed of two homologous lobes—N- and C-lobe—in which each lobe contains two Ca^2+^-binding EF-hands [21,25]. The N- and C-lobe—interconnected by a α-helix linker—are quite similar in structure, but the Ca^2+^-affinity of C-lobe is 3–10 times higher than that of N-lobe [14,21,22]. We have previously found that N- and C-lobe of CaM have lobe-specific properties in modulating Ca_V_1.2 channel [26]. However, the lobe-specific regulation of CaM on Na_V_1.1 IQ domain remains to be clarified.

CaM also acts as an activator of Ca^2+^/CaM-dependent kinase II (CaMKII). CaMKII is a multifunctional serine and threonine protein kinase activated by elevated intracellular Ca^2+^ [22,27]. Activated CaMKII and the subsequent maintenance of constitutive activity through autophosphorylation at threonine residue 286 (Thr286) are thought to play a major role in synaptic plasticity [28]. Phosphorylation of VGSCs by CaMKII dynamically regulates the expression, function, and localization of these ion channels [23,29]. A total of 70 Ser/Thr phosphorylation sites in Na_V_1.2 and 28 Ser phosphorylation sites in Na_V_1.1 have been identified in previous studies, mostly on C-terminal of α subunit [30,31,32]. However, the effect of CaMKII on CaM binding to Na_V_1.1 IQ domain has not been elucidated yet.

In the present study, we examined the binding of CaM to Na_V_1.1 IQ domain under different Ca^2+^ concentrations to demonstrate the properties of CaM binding to Na_V_1.1 IQ domain. We also prepared individual N- and C-lobe of CaM to examine the lobe-specific interactions with Na_V_1.1 IQ domain. Furthermore, we checked how CaMKII-mediated phosphorylation of the IQ domain affected the binding of CaM to the channel in order to elucidate a possible mechanism for the modulation of VGSCs by CaMKII.

## 2. Results

### 2.1. Binding of CaM to Na_V_1.1 IQ/EQ Domain

**Figure 1:**
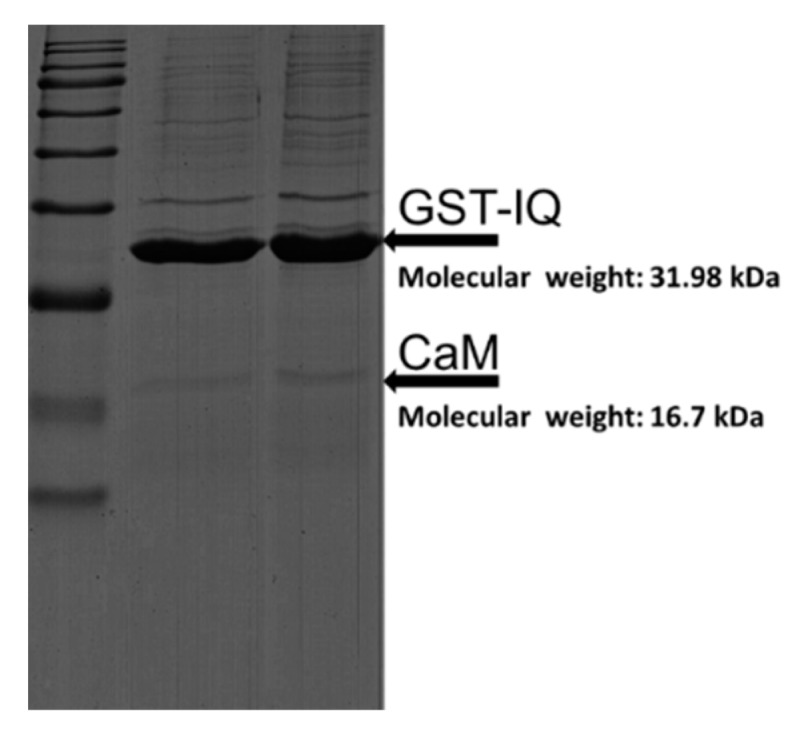
The binding of calmodulin (CaM) on GST-IQ of Na_V_1.1 with marker on the left side. The molecular weight of CaM and GST-IQ is 16.7 kDa and 31.98 kDa, respectively.

**Figure 2:**
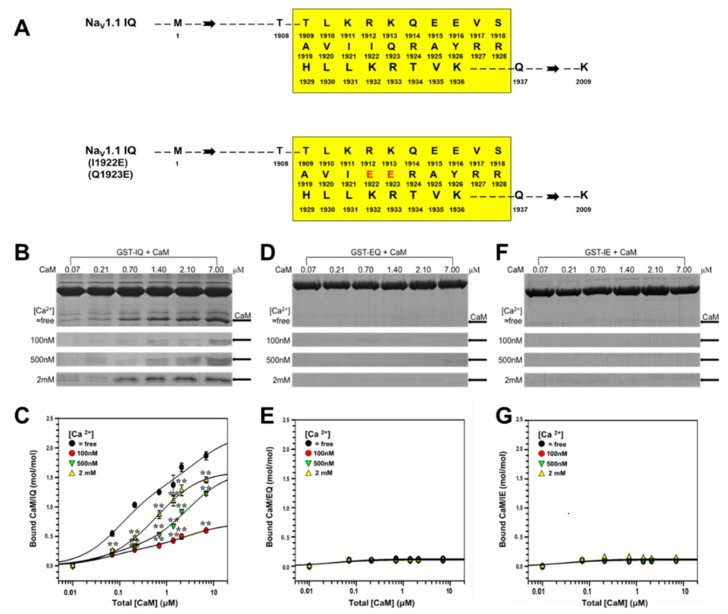
Interaction of the IQ of Na_V_1.1 and its mutant with CaM by pull-down assay. (**A**) Schematic illustrations of Na_V_1.1 IQ domain and its mutants EQ and IE. Amino acid sequences from human Na_V_1.1 IQ are presented with black letter code. The mutated amino acid of Na_V_1.1 EQ and IE is indicated with red letter. IQ, EQ and IE peptide contains amino acids from 1909 to 1936. (**B**,**D**,**F**) GST pull-down assay for the binding of CaM to IQ, EQ, or IE. (**B**,**D**,**F**) GST-fusion IQ, EQ, and IE was incubated with increasing concentrations of CaM (0.07 to 7 μm) at fixed [Ca^2+^] of ≈free, 100 nM, 500 nM, and 2 mM. Protein bands are stained by Coomassie Brilliant Blue. CaM protein bands are pointed out by arrows (**B**,**D**,**F**). Bound CaM (**C**,**E**,**G**) are plotted against total [CaM] on a molar ratio basis (CaM/IQ, EQ or IE) with mean ± S.E. (*n* = 4 for CaM). ** *p* < 0.01, compared with corresponding bindings at Ca^2+^-free conditions.

Previous research have shown Na_V_1.1 IQ domain to bind with CaM [15,29]. Therefore, we first confirmed the binding property of CaM to IQ. As shown in Figure 1, the binding of CaM to IQ was successfully detected, and the molecular weight of GST-IQ (glutathione Sepharose transferase- isoleucine and glutamine) and CaM was 31.98 and 16.7 kDa corresponding to the marker, respectively. As shown in Figure 2B, the binding of CaM to IQ was detected at ≈free, 100 nM, 500 nM, and 2 mM [Ca^2+^]. The summarized data from the densitometer analyses of replicate gels are shown in Figure 2C and Table 1. The maximal binding estimated *B*_max_ of CaM to IQ was 2.06 (*B*_max1_ + *B*_max2_), 0.66 (*B*_max1_ + *B*_max2_), 1.08 (*B*_max1_ + *B*_max2_), and 1.38 (*B*_max1_ + *B*_max2_) mol/mol (CaM/IQ) at ≈free, 100 nM, 500 nM, and 2 mM [Ca^2+^], respectively (*n* = 4), indicating that apoCaM had the highest affinity with IQ domain. However, the binding of CaM to IQ domain was in a Ca^2+^-dependent manner in the presence of Ca^2+^. The binding affinity estimated as *K*_d_ value also showed a Ca^2+^-dependent increase in the presence of Ca^2+^ (Table 1). Our data showed the binding of Ca^2+^/CaM to IQ was in a concentration-dependent and Ca^2+^-dependent manner, but apoCaM had the highest affinity to Na_V_1.1 IQ domain.

Our previous study had examined the effect of I (isoleucine)/E (glutamic acid) mutation on the IQ domain of Ca_V_1.2 on the CaM binding to this domain. We had found that the mutation completely abolished CaM binding and confirmed that I1653 in the IQ domain was important for the interaction with CaM [33]. In this study, we mutated I1922 and Q1923 in Na_V_1.1 IQ domain [^1909^TLKRKQEEVSAVIIQRAYRRHLLKRTVK^1936^] into E (Figure 2A). As shown in Figure 2D,F, the binding of CaM to EQ (I1922E) and IE (Q1923E) was diminished, confirming that I1922 (and Q1923) were the core amino acids in Na_V_1.1 IQ domain for the binding with CaM. The summarized data from the densitometer analyses of replicate gels are shown in Figure 2E,G.

### 2.2. Binding of CaM Mutants to Na_V_1.1 IQ Domain

**Figure 3:**
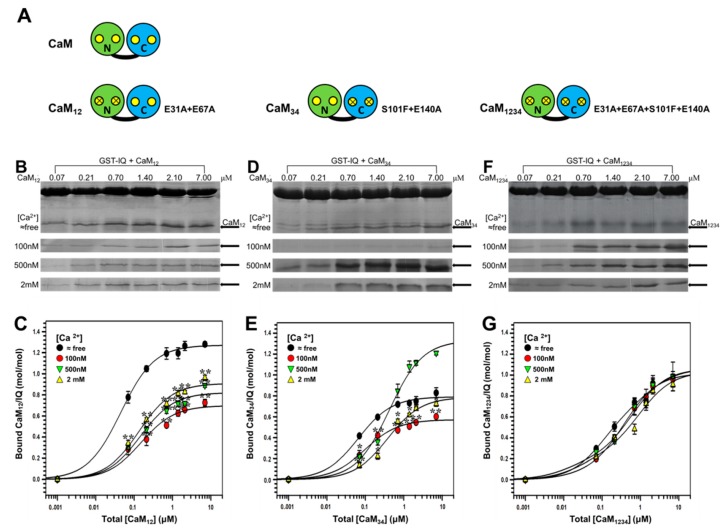
Interaction of CaM and its mutants (CaM_12_/CaM_34_/CaM_1234_) with Na_V_1.1 IQ domain by pull-down assay. (**A**) Schematic illustrations of CaM and its mutant CaM_12_, CaM_34_, CaM_1234_. Yellow circles represent normal Ca^2+^-binding sites on N/C-lobe. Yellow circles with X on it represent neutralized Ca^2+^-binding sites. Amino acid mutations are shown with red letter code. (**B**,**D**,**F**) GST pull-down assay for the binding of CaM_12_ (**B**)/CaM_34_ (**D**)/CaM_1234_ (**F**) to IQ. GST-fusion IQ was incubated with increasing concentrations of CaM_12_/CaM_34_/CaM_1234_ (0.07–7 μm) at fixed [Ca^2+^] of ≈free, 100 nM, 500 nM, and 2 mM. Protein bands were stained by Coomassie Brilliant Blue. CaM_12_/CaM_34_/CaM_1234_ protein bands are pointed out by arrows. (**C**,**E**,**G**) Bound CaM_12_ (**C**)/CaM_34_ (**E**)/CaM_1234_ (**G**) are plotted against total [CaM_12_]/[CaM_34_]/[CaM_1234_] on a molar ratio (CaM_12_ or CaM_34_ or CaM_1234_/GST-IQ) basis with mean ± S.E. (*n* = 4 for CaM_12_/CaM_34_/CaM_1234_).* *p* < 0.05, ** *p* < 0.01, compared with corresponding bindings at Ca^2+^-free conditions.

To further clarify the regulatory mechanism of CaM on Na_V_1.1 channel, we then examined the binding properties of CaM mutants to Na_V_1.1 IQ domain. This included CaM_12_ and CaM_34_ (Figure 3A) in which Ca^2+^-binding to its N- and C-lobe was eliminated, respectively, and a Ca^2+^-insensitive CaM mutant (CaM_1234_) (Figure 3A) [26]. As shown in Figure 3B,C, the binding of CaM_12_ to IQ was qualitatively similar to that of the wild-type (wt) CaM. The maximal binding estimated as *B*_max_ was 1.27, 0.70, 0.82, and 0.91 mol/mol CaM_12_/IQ (*n* = 4) at ≈free, 100 nM, 500 nM and 2 mM Ca^2+^, respectively, showing an obvious Ca^2+^ dependence in the presence of Ca^2+^ (Table 1). It is interesting to note that the *B*_max_ of CaM_12_ at ≈free Ca^2+^ is ~30% was greater than that at 2 mM Ca^2+^, suggesting that like wt CaM, CaM_12_ has the highest affinity for IQ in the absence of Ca^2+^.

Next, we examined the binding of CaM_34_ to IQ domain. As shown in Figure 3D,E, the binding of CaM_34_ to IQ was also concentration-dependent. The parameters obtained (Table 1) revealed that the maximal binding estimated as *B*_max_ was 0.79, 0.57, 1.33, and 0.79 mol/mol CaM_34_/ IQ (*n* = 4) at ≈free, 100 nM, 500 nM, and 2 mM Ca^2+^, respectively. It was noted that this profile of [Ca^2+^] dependence was different from those of wt CaM and CaM_12_.

Furthermore, we examined the binding of CaM_1234_ to Na_V_1.1 IQ domain. As shown in Figure 3F, the Ca^2+^ dependence was totally diminished because of the mutation in four Ca^2+^ binding sites. As shown in Figure 3G and Table 1, the *B*_max_ were 1.06, 1.02, 1.06, and 1.03 mol/mol (CaM_1234_/IQ) at ≈free, 100 nM, 500 nM, and 2 mM [Ca^2+^], respectively. The values of *B*_max_ and *K*_d_ were not significantly different among different Ca^2+^ concentrations, confirming the Ca^2+^-insensitive nature of CaM_1234_. Our data showed that functional Ca^2+^ binding sites of either N- or C-lobe were required for Ca^2+^ dependency in CaM binding to Na_V_1.1 IQ domain.

### 2.3. Binding of Individual N-Lobe or C-Lobe of CaM to Na_V_1.1 IQ Domain

**Figure 4:**
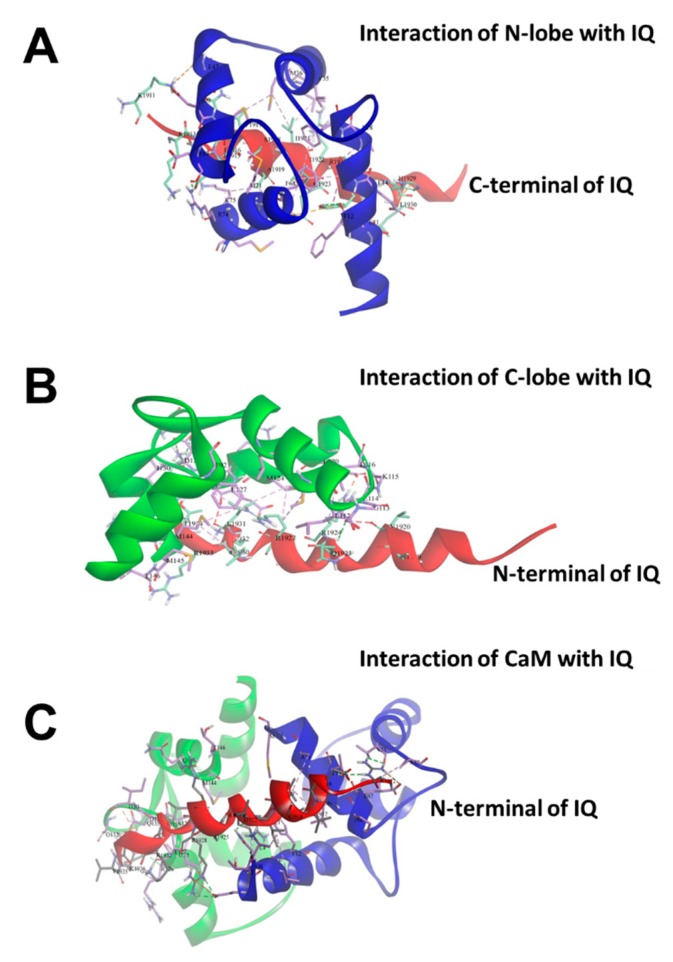
Interaction of the N-lobe/C-lobe of CaM with Na_V_1.1 IQ domain by docking experiment. (**A**) The lowest interaction energy pose for the interaction of N-lobe of CaM with Na_V_1.1 IQ domain; (**B**) The lowest interaction energy pose for the interaction of C-lobe of CaM with Na_V_1.1 IQ domain; and (**C**) The lowest interaction energy pose for the interaction of the N-lobe and C-lobe of CaM with Na_V_1.1 IQ domain. **Red** ribbon—Na_V_1.1 IQ; **blue** ribbon—N-lobe; **green** ribbon—C-lobe. Interaction residues and nonbond interactions are indicated as well.

In order to study the effect of specific lobe of CaM on the Na_V_1.1 IQ, we first computationally investigated the interactions between N-lobe or C-lobe of CaM and Na_V_1.1 IQ domain using Discovery Studio 2017. As shown in Figure 4A,B, the ZDock score and E_RDock for the optimal N-lobe orientation docking into Na_V_1.1 IQ domain were 11.28 and −23.31 kcal/mol, respectively, whereas these parameters for the best interaction of C-lobe with IQ domain were 10.66 and −26.21 kcal/mol, respectively. In addition, as shown in Figure 4C, when the N- and C- lobe were docked together into the Na_V_1.1 IQ domain, the best ZDock score and lowest E_RDock were 12.78 and −30.72 kcal/mol, respectively.

**Figure 5:**
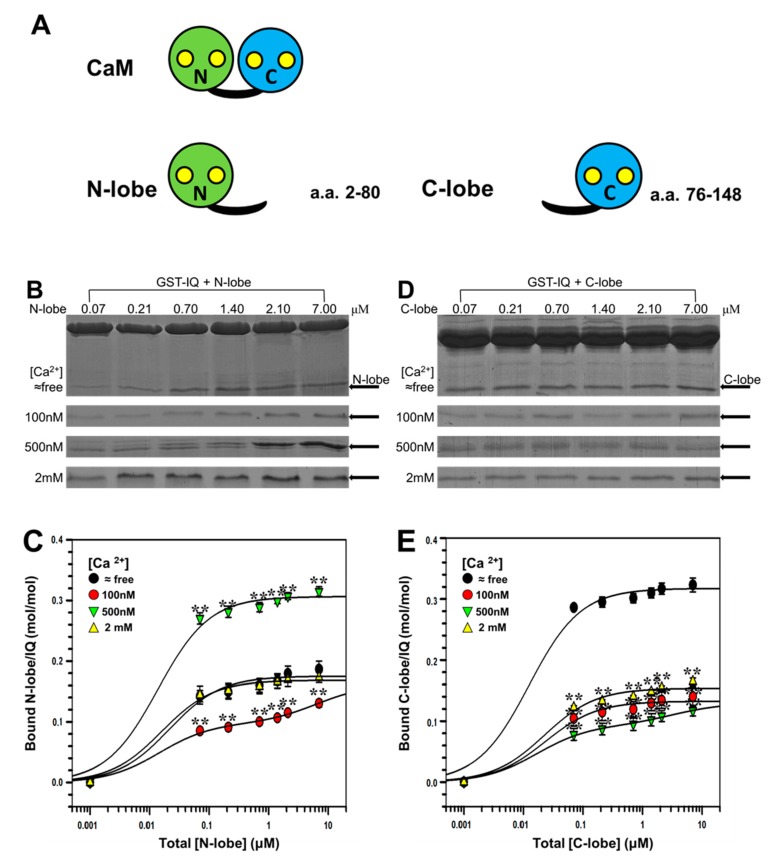
Interaction of the N-lobe/C-lobe of CaM with Na_V_1.1 IQ domain by pull-down assay. (**A**) Schematic illustrations of CaM and its truncated protein N-lobe (green), C-lobe (blue). N-lobe peptide contains amino acids from 2 to 80 and C-lobe peptide contains amino acids from 76 to 148. Yellow circles represent normal Ca^2+^-binding sites on N/C-lobe; (**B**,**D**) GST pull-down assay for the binding of N-lobe (**B**) or C-lobe **(D**) to IQ domain. GST-fusion IQ was incubated with increasing concentrations of N-lobe or C-lobe (0.07 to 7 μm) at fixed [Ca^2+^] of ≈free, 100 nM, 500 nM, and 2 mM. Protein bands were stained by Coomassie Brilliant Blue. N-lobe or C-lobe bands are pointed out by arrows; and (**C**,**E**) Bound N-lobe (**C**) or C-lobe (**E**) are plotted against total [N-lobe] or [C-lobe] on a molar ratio basis (N-lobe or C-lobe/GST-IQ) with mean ± S.E. (*n* = 4). ** *p* < 0.01, compared with corresponding bindings at Ca^2+^-free conditions.

Next, we further examined the bindings of individual (truncated) N- and C-lobe of CaM (Figure 5A) to IQ domain under different Ca^2+^ concentrations by pull-down assay. As shown in Figure 5B,C and Table 2, the maximal bindings estimated as *B*_max_ for N-lobe were 0.18, 0.16, 0.31, and 0.17 mol/mol (N-lobe/IQ) at ≈free, 100 nM, 500 nM, and 2 mM [Ca^2+^], respectively (*n* = 4). Thus the profile of [Ca^2+^] dependence of N-lobe was similar to that of CaM_34_.

We then examined the binding of C-lobe to IQ domain. As shown in Figure 5D, like wt CaM, C-lobe also had the highest affinity with IQ at ≈free [Ca^2+^]. The maximal binding presented by *B*_max_ were 0.32, 0.13, 0.13, and 0.15 mol/mol (C-lobe/IQ) at ≈free, 100 nM, 500 nM, and 2 mM [Ca^2+^], respectively (*n* = 4) (Figure 5E and Table 2). Thus, the profile of [Ca^2+^] dependence of C-lobe was similar to those of wt CaM and CaM_12_.

The parameters (Table 2) revealed that binding of both N- and C-lobe to IQ was also in a Ca^2+^-dependent manner. *K*_d_ of N-lobe was lower than that of C-lobe in the presence of Ca^2+^ ([Ca^2+^] ≥ 100 nM), whereas *K*_d_ of C-lobe was lower than that of N-lobe in the absence of Ca^2+^. Additionally, *B*_max_ of N-lobe was higher than that of C-lobe in the presence of Ca^2+^, whereas *B*_max_ of C-lobe was higher than that of N-lobe in the absence of Ca^2+^, indicating that C-lobe was the predominant domain in apoCaM interacting with Na_V_1.1 IQ domain, and N-lobe was the predominant domain in Ca^2+^/CaM interacting with Na_V_1.1 IQ domain.

### 2.4. The Effect of CaMKII on CaM Binding to Na_V_1.1 IQ Domain

**Figure 6:**
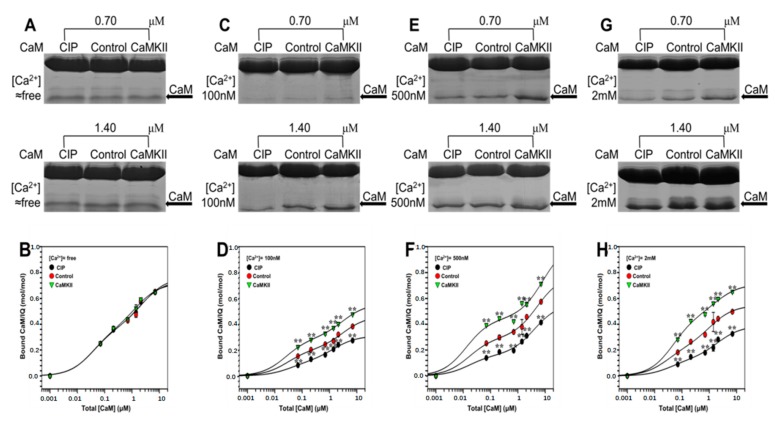
Regulation of CaMKII on CaM binding to Na_V_1.1 IQ domain by pull-down assay. (**A**,**C**,**E**,**G**) GST pull down assay for the binding of CaM with CaMKII-meditated phosphorylation IQ. GST-fusion IQ was phosphorylated by CaMKII, then incubated with increasing concentration of CaM (0.07 to 7 μm) at fixed [Ca^2+^] of ≈free, 100 nM, 500 nM, and 2 mM. Protein bands were stained by Coomassie Brilliant Blue. CaM bands are pointed out by arrows. (**B**,**D**,**F**,**H**) Bound CaM are plotted against total [CaM] on a molar ratio basis (CaM/GST-IQ) with mean ± S.E. (*n* = 4). ** *p* < 0.01, compared with corresponding bindings at control conditions.

In the previous study, several CaMKII-mediated phosphorylation sites on Na_V_1.1 have been identified [29]. To clarify the modulation of CaMKII on Na_V_1.1, we further checked the effect of CaMKII on CaM binding to Na_V_1.1 IQ domain. As shown in Figure 6A,B, the binding of apoCaM to IQ barely changed after phosphorylation at ≈free [Ca^2+^] compared to that in the presence of Ca^2+^, suggesting that phosphorylation of IQ domain by CaMKII had little effect on its binding with apoCaM. By contrast, the binding of Ca^2+^/CaM to IQ was increased in the phosphorylated IQ, indicating that the effect of CaMKII on the CaM binding to Na_V_1.1 IQ domain could exert its regulation only in the presence of Ca^2+^ (Figure 6C,E,G). The parameters obtained (Figure 6D,F,H and Table 3) revealed that CaMKII-mediated phosphorylation increased the binding of CaM to IQ, while dephosphorylation by CIP decreased the affinity of CaM with IQ. In addition, *K*_d_ and *B*_max_ showed that an increased binding of Ca^2+^/CaM to the channel at higher [Ca^2+^] was observed compared to that at 100 nM [Ca^2+^].

**Figure 7:**
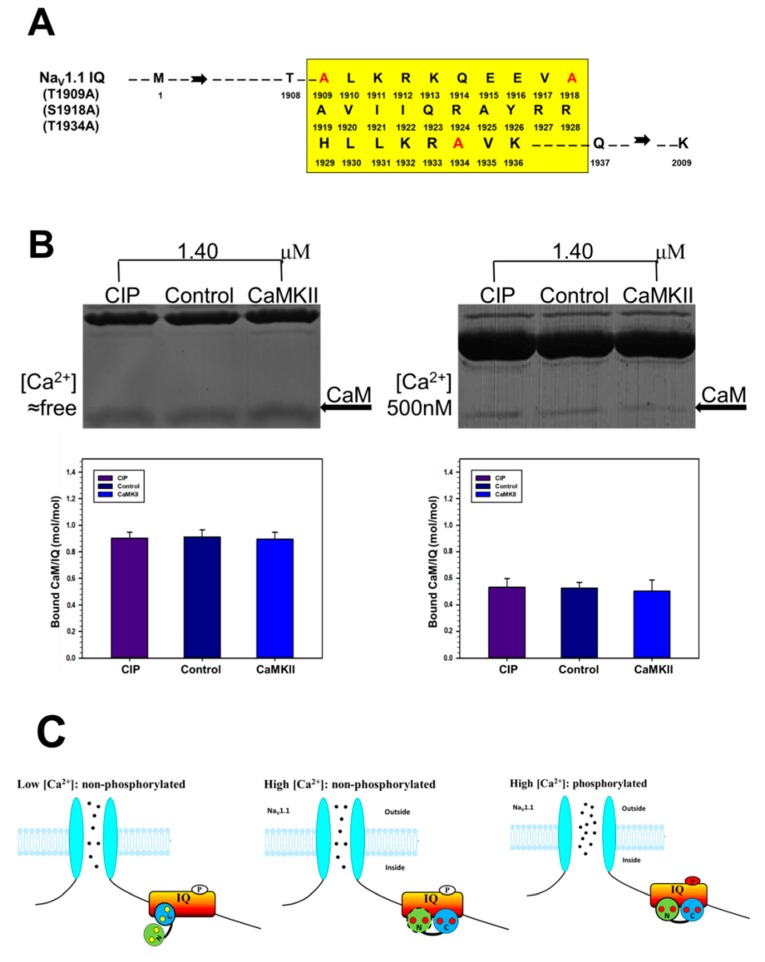
Neutralized CaMKII-mediated phosphorylation sites on Na_V_1.1 IQ domain. (**A**) Schematic illustrations of mutated Na_V_1.1 IQ domain with three potential CaMKII-mediated phosphorylation sites, T1909A, S1918A, and T1934A. The mutated amino acids are presented by red letter code; (**B**) GST pull-down assay for the binding of CaM to mutated Na_V_1.1 IQ. GST-fusion IQ was incubated with fixed concentration of CaM (1.4 μm) at fixed [Ca^2+^] of ≈free and 500 nM. Protein bands were stained by Coomassie Brilliant Blue. CaM protein bands are pointed out by arrows. (**B**) Bound CaM are plotted against total [CaM] on a molar ratio basis (CaM/IQ) with mean ± S.E. (*n* = 4 for CaM); and (**C**) Schematic illustrations of a hypothetical model for the modulation of Ca^2+^/CaM/CaMKII on Na_V_1.1. At low [Ca^2+^], a nonphosphorylated state of IQ domain, C-lobe is the predominant domain for apoCaM binding to IQ domain, and the binding of apoCaM to Na_V_1.1 IQ is not affected by CaMKII. At high [Ca^2+^], but a nonphosphorylated state of IQ domain, N-lobe becomes the predominant domain since some of CaM binds with Ca^2+^. Phosphorylation of IQ by CaMKII modulates the binding of Ca^2+^/CaM to the channel. Meanwhile, channel activity is maintained to the basal level. At high [Ca^2+^], a phosphorylated state of IQ domain, N-lobe is the predominant domain for Ca^2+^/CaM binding to IQ domain. Meanwhile, the effect of CaMKII phosphorylation is further promoted, leading to an increased binding of Ca^2+^/CaM to the channel compared to that at 80–100 nM [Ca^2+^]. The channel activity would be also increased at high [Ca^2+^] compared to that at low [Ca^2+^]. Red and black circles represent Ca^2+^ and Na^+^, respectively. Red oval with a P on it represents activated phosphorylation site.

In a previous study, one CaMKII-mediated phosphorylation site S1920 on Na_V_1.5 IQ domain had been identified. In addition, CaMKII is a basophilic protein kinase belonging to the Ca^2+^/CaM dependent superfamily of serine/threonine kinases (Herren et al., 2015). Thus, we mutated three potential CaMKII-mediated phosphorylation sites—T1909, S1918, and T1934—into A (Figure 7A), then Na_V_1.1 IQ domain was treated with CaMKII and CIP. As shown in Figure 7B, under free [Ca^2+^] condition, there was no significant difference in the CaM binding to the IQ domain between the phosphorylated and dephosphorylated peptides as well as 500 nM [Ca^2+^] condition, indicating that CaMKII facilitated the binding of Ca^2+^/CaM to Na_V_1.1 IQ domain due to one or several phosphorylation sites in T1909, S1918, and T1934 of Na_V_1.1 IQ domain.

## 3. Materials and Methods

### 3.1. cDNA Construction and Site-Directed Mutagenesis

The cDNA corresponding to the IQ domain of Na_V_1.1 (IQ, amino acids 1909–1936) was generated by PCR using the cDNA of human Na_V_1.1 as the template. The primers were designed using VectorNTI software. The human CaM and its truncated proteins—N-lobe (a.a. 2–80) and C-lobe (a.a. 76–148)—were subcloned from HEK293 cells [21,34,35]. The CaMKII mutant T286D (CaMKIIT286D), which is a constitutively active type of CaMKII in the absence of CaM and Ca^2+^, was created with rat CaMKIIα cDNA as a template [36]. Mutants including IQ mutant (I1922E, Q1923E), potential CaMKII phosphorylation sites mutant (T1909A + S1918A + T1934A), CaM_12_ (E31A + E67A), CaM_34_ (S101F + E140A), and CaM_1234_ (E31A + E67A + S101F + E140A) were constructed by site-directed mutagenesis using a Quickchange™ kit (QIAGEN) [37,38]. The above DNAs were individually ligated into pGEX-6P-1 H320 expression vectors (GE Biosciences, New York, NY, USA).

### 3.2. Expression and Purification of Recombinant GST Fusion Peptides

The above described vectors were transformed into *Escherichia coli* BL21 (DE3) to express the target peptides as glutathione-*S*-transferase (GST) fusion proteins. The bacteria were cultured in LB liquid medium at 37 °C overnight until an OD600 of 0.8–1.0. Then, the bacteria were induced with 1 mM isopropyl-1-thio-β-d-galactopyranoside (IPTG) and continued incubating for 4 h at 37 °C before harvesting. The ultrasonic technique was used to harvest GST-fusion peptides. Then, the fusion peptides were purified using Glutathione Sepharose 4B beads (GS-4B, GE Healthcare, New York, NY, USA). The GST regions of CaM and its mutants were cleaved with PreScission Protease (GE Healthcare). GST-IQ, CaM, and its mutants were quantified by Enhanced BCA Protein Assay Kit with BSA as standard with correction factors 1.25 (GST-IQ), 1.69 (CaM and its full-length mutants), 0.82 (N-lobe of CaM), and 0.82 (C-lobe of CaM).

### 3.3. GST Pull-Down Assay

GST-fusion IQ or its mutant (2–4 μg) was immobilized on GS-4B and incubated in 300 μL of Tris buffer (consisting of 150 mM NaCl, 50 mM Tris, and pH 8.0 adjusted by HCl) with increasing concentrations of CaM or its mutants (0.07, 0.21, 0.7, 1.4, 2.1, 7.0 μm) for 4 h at 4 °C under agitation in the presence of different Ca^2+^ concentrations ([Ca^2+^] ≈ free, 100, 500, and 2 mM). The [Ca^2+^] was calculated with MaxChelator (http://maxchelator.stanford.edu/index.html). Then, the reaction systems were gently washed twice with the same buffer. Bound CaM (or its mutant) and IQ (or EQ) were resuspended in 5× SDS-PAGE loading buffer and resolved in 15% SDS-PAGE gels. Proteins were stained by Coomassie brilliant blue R (CBB). Protein bands in the SDS-PAGE gels were digitized by the Photoshop software (Adobe, San Jose, CA, USA), and the grey level was quantified by Image J software (NIH, Bethesda, MD, USA) [34,35,36,37]. The optical density values were converted to protein contents using respective correction factors (see below).

The GST-fusion IQ (for control) immobilized to GS-4B beads (40 μL) was phosphorylated in an assay reaction (0.4 μm CaMKIIT286D) in Tris buffer containing 1 mM MgCl_2_ and 1 mM Na_2_ATP for 30 min at 30 °C. The reaction was then terminated with the addition of 1× SDS sample buffer and gently washed twice. The dephosphorylation was achieved by adding 5 U/mL calf intestinal alkaline phosphatase (CIP; New England Biolabs, Ipswich, MA, USA) into the reacting mixtures incubating at 37 °C for 30 min. CIP is a nonspecific phosphorylase that commonly exists in calf intestinal mucosa. The reaction was terminated by the addition of same Tris buffer and gently washed twice.

### 3.4. Computational Docking

A homology model of Na_V_1.1 IQ was constructed based upon the solved crystal structure of IQ motif of Na_V_1.2 (PDB # 2KXW) [15] using the Create Homology Model tool in Discovery Studio 2017 (BIOVIA, Boston, MA, USA). The N- and C-lobe of CaM were derived from the crystal structure of Ca^2+^/CaM-Ca_V_1.2 IQ domain complex (PDB # 3DVE) [39]. Docking studies between Na_V_1.1 IQ and CaM N- or C-lobe were performed in Discovery Studio using the Dock Proteins protocols. The ZDOCK protocol was used for docking the IQ motif of Na_V_1.1 to N- or/and C-lobe of CaM and, subsequently, the RDOCK protocol was applied for further refinement of the 10 best-docked poses. For individual interactions, docking results are displayed as solid ribbons of 1 solution with the lowest RDOCK interaction energy.

### 3.5. Statistical Analysis

Quantified grey level was converted into molar quantities according to the mass of GST-IQ, CaM, N-lobe, and C-lobe of CaM. We found that relative optical densities of the same amount of these proteins on the gel in reference to BSA were 0.80, 0.59, 1.21, and 1.22 respectively, from which the correction factors were determined as 1.25, 1.69, 0.82, and 0.82, respectively. Curve-fitting of the total bound ligand (CaM and its mutants) was performed with the software SigmaPlot 12.0 (version 12, Sigma-Aldrich, Beijing, China). Bound ligand (*y*) was fitted with the following Hill’s equation for the one-site fitting model: *y* = *B*_max_·*x*/(*K*_d_ + *x*). For the two-sites model, a sum of two Hill’s equations was integrated to assume independent binding: *y* = *B*_max1_·*x*/(*K*_d1_ + *x*) + *B*_max2_·*x*/(*K*_d2_ + *x*), where *B*_max1_, *B*_max2_, *K*_d1_, and *K*_d2_ represents total *B*_max_ and *K*_d_, respectively; *x* is the concentration of free ligand; *K*_d_ is the apparent dissociation constant; and *B*_max_ is the maximum binding for each binding site. We chose either one-site model or two-sites model based on the higher value of *R*^2^. Hill’s coefficient of 1.0 was assumed. Total concentration of ligand was assumed as an approximate of free ligand. The data are presented as mean ± S.E. (*n* = 4). The SPSS 22.0 software (version 22, Sigma-Aldrich, Beijing, China) was used to evaluate the statistical significance, and *p* < 0.05 by hypothesis test was considered statistically significant.

## 4. Discussion

Our study was aimed at clarifying the molecular mechanism underlying the modulation of Ca^2+^/CaM/CaMKII on Na_V_1.1 channel, which is a key issue in understanding the regulatory mechanism of VGSCs. Although the modulation of VGSCs has been a hot spot in ion channel research, the present study examined for the first time the effects of Ca^2+^ and CaM on Na_V_1.1 channel in a wide range of [Ca^2+^] using CaM mutants.

CaM is the most important Ca^2+^ binding protein and is involved in the regulation of numerous Ca^2+^-dependent pathways. Its function and structure depend strongly on Ca^2+^ concentration [40,41]. In our research, we checked the effect of different Ca^2+^ concentrations on the binding of CaM to IQ domain of Na_V_1.1. Our data showed that the binding of CaM to Na_V_1.1 IQ domain was Ca^2+^- and concentration-dependent. Full-length CaM switches from a simple folding structure at lower [Ca^2+^] to a rich and complex folding behavior at high [Ca^2+^] [41]. Accordingly, similar Ca^2+^-dependent conformational changes in CaM between Na_V_1.2 and Na_V_1.5 have previously been reported [8,40]. Our results showed that the binding of CaM to Na_V_ 1.1 IQ was Ca^2+^-dependent, which reflects the Ca^2+^-dependent conformational change of CaM.

CaM often modulates target molecules only upon conversion to its Ca^2+^-bound form. However, apoCaM binding in itself markedly promotes opening of voltage-gated calcium channels (VGCCs) [9,11,34,41]. VGSCs have also been suggested to adopt a similar modulatory principle [9,16]. Our present data showed that the binding of Ca^2+^/CaM to Na_V_1.1 IQ was dramatically decreased compared to that of apoCaM, implying that apoCaM promotes activity of Na_V_1.1 channels. On the contrary, studies on Ca_V_1.2 indicated that the binding of apoCaM to the channel was significantly smaller compared to that of Ca^2+^/CaM [21,37,42]. This may suggest that CaM binds to different channels in a channel-specific manner, meaning the detailed mechanism of CaM regulation may need to be considered on a channel-specific basis.

It has been reported that regulatory effect of CaM on VGCCs is lobe-specific, and N- and C-lobe of CaM have distinct roles in the regulation of VGCCs [7,40,43]. In addition, a single Ca^2+^/CaM bridges the C-terminal IQ motif of Na_V_1.5 to the DIII-IV linker via individual N- and C-lobes, respectively. C-lobe binds to IQ (N-lobe is free) at low [Ca^2+^], whereas at high [Ca^2+^], N-lobe binds to IQ (lobe switching) and C-lobe binds to III-IV linker, resulting in depolarization of the inactivation curve [44]. Furthermore, the most prominent Ca^2+^-dependent conformational change is the interaction between the calcified N-lobe of CaM and the Na_V_ IQ domain; the CaM C-lobe remains Ca^2+^-free even in millimolar Ca^2+^, remains bound to the IQ motif, and retains its semi-open conformation. Despite similar Ca^2+^-dependent conformational changes between the Na_V_1.2 and Na_V_1.5 complexes, the functional effects are isoform-specific, while their mechanistic bases are not clear [40]. However, the lobe specificity of CaM modulation of Na_V_1.1 have not been demonstrated. In this study, we applied individual N- and C-lobe of CaM to further explore the role of individual lobes in binding to Na_V_1.1 IQ domain. The affinity of IQ for C-lobe binding was higher than that of N-lobe in the absence of Ca^2+^, whereas the affinity of IQ for N-lobe binding was higher than that of C-lobe in the presence of Ca^2+^, indicating distinct responses at different [Ca^2+^]. Thus, the property difference between the two lobes of CaM might endow the Ca^2+^-dependent and lobe-specific modulation of Na_V_1.1 channel. However, we still do not know the reason why CaM binding to the IQ domain is the largest at free Ca^2+^ and smaller at 100 nM Ca^2+^. We speculate that C-lobe of CaM might be at least partially occupied with Ca^2+^ at 100 nM Ca^2+^. In this scenario, the weaker binding of CaM to the IQ domain would be explained by the fact that the IQ domain has lower affinity for Ca^2+^/C-lobe than for Ca^2+^-free C-lobe. This point may be supported by the CaM_12_ and CaM_34_ experiments (Figure 3B–E). In the CaM_12_ experiment, the binding property to the IQ at free and 100 nM Ca^2+^ was similar to that of wild-type CaM, while this property was less pronounced in the CaM_34_ experiment.

One of the most intriguing findings of our research is that CaMKII-mediated phosphorylation of Na_V_1.1 IQ domain increased binding of CaM to the channel. It has become recognized that both the expression and function of VGSCs is under tight control of protein phosphorylation by protein kinases [45]. CaMKII activated by Ca^2+^/CaM maintains activity of VGCCs [36], CaMKII has emerged as a critical regulator of Na_V_1.5, and multiple CaMKII-mediated phosphorylation sites have been identified on Na_V_1.5, including S1920 and S1925, which are located on IQ domain and noncanonical CaMKII sites. [29,46]. In addition, CaMKII-enhanced I_NaL_ positively shifts inactivation curve of Na_V_1.2 epileptic mutant (Q54) [23,47]. Based on the structural homology of Na_V_1.1 and Na_V_1.5, we treated Na_V_1.1 IQ domain with CaMKII and CIP to examine a possible regulation of Na_V_1.1 by CaMKII. Under low [Ca^2+^] condition, we found there was no significant difference in the CaM binding to the IQ domain between the phosphorylated and dephosphorylated peptides. One possible reason for this may be that the phosphorylation of Na_V_1.1 IQ might not affect the conformation of the apoCaM binding region, which interacts mainly with C-lobe of CaM. However, in the presence of Ca^2+^ CaMKII-mediated phosphorylation of IQ increased the binding of CaM to IQ domain, while CIP-mediated dephosphorylation of IQ decreased the binding of CaM. Thus, it is possible that Ca^2+^/CaM binding region interacts mainly with N-lobe of CaM, which might be different from the apoCaM binding region. Our data has shown that CaMKII regulates the binding of Ca^2+^/CaM to Na_V_1.1 IQ domain due to one or several phosphorylation sites in T1909, S1918, and T1934 of Na_V_1.1 IQ domain, indicating that CaMKII-mediated phosphorylation of Na_V_1.1 affects CaM binding to Na_V_1.1. It is therefore speculated that CaMKII-mediated phosphorylation of Na_V_1.1 IQ domain might change the conformation of IQ domain, leading to increased binding of Ca^2+^/CaM to Na_V_1.1.

A previous study had shown that CaM overexpression in HEK1.1 cells increases the peak current of Na_V_1.1 in a calcium-dependent manner [12]. Our previous study has also demonstrated that neuronal VGSC activity is modulated by CaM in a concentration-dependent manner in normal and low Mg^2+^ condition [13]. Thus, we propose the following hypothetical model (Figure 7C) for the modulation of Ca^2+^/CaM/CaMKII on Na_V_1.1 based on our present study and other studies [12,13]: At low [Ca^2+^]—a nonphosphorylated state of Ca^2+^ concentration—C-lobe is the predominant domain for apoCaM binding to IQ domain, and the binding of apoCaM to Na_V_1.1 IQ is not affected by CaMKII. At high [Ca^2+^] but at a nonphosphorylated state of IQ domain, N-lobe becomes the predominant domain since some of the CaM binds with Ca^2+^. Phosphorylation of IQ by CaMKII modulates the binding of Ca^2+^/CaM to the channel. Meanwhile, channel activity is maintained to the basal level at 80–100 nM [Ca^2+^]. At high [Ca^2+^]—a phosphorylated state of IQ domain—N-lobe is the predominant domain for Ca^2+^/CaM binding to IQ domain. Meanwhile, the effect of CaMKII-mediated phosphorylation is further promoted, leading to an increased binding of Ca^2+^/CaM to the channel compared to that at 80–100 nM [Ca^2+^]. The channel activity will also be increased at high [Ca^2+^] compared to that at 80–100 nM [Ca^2+^].

In summary, we found that the binding of Ca^2+^/CaM to IQ was Ca^2+^- and concentration-dependent, and apoCaM more preferentially binds to Na_V_1.1 IQ domain than Ca^2+^/CaM. In addition, C-lobe of CaM is the predominant domain in apoCaM binding to Na_V_1.1 IQ domain, whereas N-lobe of CaM is the predominant domain in Ca^2+^/CaM binding to Na_V_1.1 IQ domain. In addition, CaMKII-mediated phosphorylation increases the binding of Ca^2+^/CaM to Na_V_1.1 IQ domain due to one or several phosphorylation sites in T1909, S1918, and T1934 of Na_V_1.1 IQ domain. Our data provides novel mechanisms for the modulation of Na_V_1.1 by the Ca^2+^/CaM/CaMKII axis. For the first time, we uncover the effect of Ca^2+^, lobe-specificity, and CaMKII on CaM binding to Na_V_1.1.

## Figures and Tables

**Table 1:** Parameters for the bindings of CaM and its mutants to Na_V_1.1 IQ domain.

Parameters	CaM				CaM_12_				CaM_34_				CaM_1234_			
	[Ca^2+^] ≈ Free	100 (nM)	500 (nM)	2 (mM)	[Ca^2+^] ≈ Free	100 (nM)	500 (nM)	2 (mM)	[Ca^2+^] ≈ Free	100 (nM)	500 (nM)	2 (mM)	[Ca^2+^] ≈ Free	100 (nM)	500 (nM)	2 (mM)
*B*_max1_ (mol/mol)	1.2296	0.2796	0.2505	0.1258	1.272	0.6985	0.822	0.9093	0.7911	0.5719	1.3305	0.7875	0.6932	0.061	0.1927	0.2423
*K*_d1_ (μM)	0.1036	0.063	0.0433	0.0682	0.0453	0.1533	0.1481	0.1347	0.0665	0.0978	0.4231	0.3865	0.1023	0.1684	0.0132	0.0297
*B*_max2_ (mol/mol)	0.8282	0.3838	0.8312	1.2559									0.3712	0.9562	0.8682	0.792
*K*_d2_ (μM)	4.5817	2.9054	1.5099	0.5589									1.632	0.3822	0.6179	0.952
*R* ^2^	0.9824	0.9861	0.9745	0.9955	0.9978	0.9725	0.9762	0.9858	0.9935	0.9841	0.9908	0.9879	0.9902	0.9984	0.9857	0.9783
*p*		0.004	0.002	0.002		0.001	0.001	0.001		0.001	0.296	0.017		0.104	0.105	0.031

**Table 2:** Parameters for the bindings of truncated CaM to Na_V_1.1 IQ domain.

Parameters	N-Lobe				C-Lobe			
	[Ca^2+^] ≈ Free	100 (nM)	500 (nM)	2 (mM)	[Ca^2+^] ≈ Free	100 (nM)	500 (nM)	2 (mM)
*B*_max1_ (mol/mol)	0.0893	0.0973	0.1532	0.085	0.1483	0.0928	0.0675	0.0784
*K*_d1_ (μM)	0.0214	0.0144	0.0137	0.016	0.0124	0.0167	0.0231	0.0225
*B*_max2_ (mol/mol)	0.0859	0.0645	0.1531	0.0836	0.1691	0.0369	0.0652	0.0755
*K*_d2_ (μM)	0.0214	6.3762	0.0137	0.016	0.0124	3.3088	0.0231	0.0225
*R* ^2^	0.9788	0.9937	0.9879	0.9891	0.9873	0.9954	0.9832	0.9817
*p*		0.001	0.001	0.198		0.001	0.001	0.001

**Table 3:** Parameters for the bindings of CaM to phosphorylated Na_V_1.1 IQ domain.

Parameters	‘Phosphorylation ([Ca^2+^] ≈ Free)			Phosphorylation ([Ca^2+^] = 100nM)			Phosphorylation ([Ca^2+^] = 500nM)			Phosphorylation ([Ca^2+^] = 2mM)		
	CIP	Control	CaMKII	CIP	Control	CaMKII	CIP	Control	CaMKII	CIP	Control	CaMKII
*B*_max1_ (mol/mol)	0.3662	0.3753	0.3623	0.1141	0.2063	0.2925	0.1624	0.2952	0.4425	0.1308	0.2304	0.4709
*K*_d1_ (μM)	0.0371	0.0376	0.0361	0.0365	0.029	0.0251	0.0163	0.0163	0.013	0.0383	0.0283	0.0473
*B*_max2_ (mol/mol)	0.3713	0.3862	0.3572	0.1967	0.2572	0.2667	0.3969	0.4848	0.5894	0.2564	0.3163	0.24
*K*_d2_ (μM)	2.0347	2.4926	1.4381	1.4219	3.0904	3.026	3.9451	5.0388	8.1743	2.006	1.1043	1.9515
*R* ^2^	0.9955	0.9891	0.9951	0.9944	0.9962	0.9987	0.991	0.9939	0.9789	0.9861	0.9937	0.9954
*p*	0.91		0.175	0.001		0.001	0.001		0.001	0.002		0.001

Data from the GST pull-down assay shown in Figure 1, Figure 2, Figure 3 and Figure 4 analyzed with single or double Hill’s equations at [Ca^2+^] from ≈free to 2 mM. *K*_d_—apparent dissociation constants; *B*_max_—the maximum bindings; *R*^2^—coefficient of determination; *p*—significance probability.
